# Exploring the Comparative Impact of Red and White Meat on Cardiovascular Diseases: A Global Cross‐Sectional Ecological Study

**DOI:** 10.1002/hsr2.70990

**Published:** 2025-06-30

**Authors:** Wenpeng You, Shuhuan Feng, Frank Donnelly

**Affiliations:** ^1^ Heart and Lung, Royal Adelaide Hospital Adelaide Australia; ^2^ Adelaide Nursing School, The University of Adelaide Adelaide Australia; ^3^ Adelaide Medical School The University of Adelaide Adelaide Australia; ^4^ School of Nursing and Midwifery Western Sydney University Sydney Australia; ^5^ China Organic Food Certification Center Beijing China; ^6^ School of Nursing Murdoch University Murdoch Australia

**Keywords:** cardiovascular disease, red meat, saturated fat, white meat

## Abstract

**Background:**

The impact of white meat on cardiovascular diseases (CVDs) is rarely reported, while red meat has been extensively associated with CVDs. This phenomenon is worth exploring, considering that there is no substantial difference in composition between red meat and white meat.

**Methods:**

Country‐specific data were extracted from United Nations agencies to analyse and compare the statistical roles of red meat and white meat in contributing to CVD incidence. Ageing, socioeconomic status, obesity, and urbanization were included as confounders in four data analysis models: bivariate correlations (Pearson's *r* and nonparametric), partial correlation, and stepwise linear regression.

**Results:**

Globally, both red meat and white meat showed significant correlations with CVD incidence in bivariate analyses. However, when adjusting for confounders and the effect of red meat, red meat consistently maintained a significant association with CVD incidence, whereas the correlation for white meat became weak or negligible. These findings suggest that the apparent association between white meat consumption and CVD may be largely due to confounding by red meat intake.

**Conclusions:**

Our analyses imply that red meat consumption exhibits a significantly stronger correlation with CVD incidence than white meat consumption. Although both meat types show significant associations in bivariate analyses, the association for white meat becomes negligible after adjusting for red meat intake and other confounding factors. This suggests that the adverse effects observed in studies that consider white meat consumption may be largely attributable to concurrent red meat consumption, rather than reflecting an independent risk from white meat.

## 1 Introduction

Cardiovascular diseases (CVD) remain the leading cause of morbidity and mortality worldwide, placing a substantial burden on public health and economies. Among the factors contributing to CVD risk, dietary habits are critical. Over the past decades, a substantial body of epidemiological research has consistently linked red meat consumption to an increased risk of cardiovascular outcomes. Numerous studies have demonstrated that diets high in red meat are associated with higher CVD mortality and incidences of ischemic heart disease, and stroke [1, 2, 3, 4, 5]. Proposed mechanisms for this relationship include the high content of saturated fats, cholesterol, L‐carnitine, and heme iron found in red meat, all of which may promote atherosclerosis and systemic inflammation.

In contrast, the health effects of white meat consumption remain less thoroughly investigated and are subject to ongoing debate. Some studies suggest that white meat may have a neutral or even protective association with CVD risk [2, 6, 7], leading to the suggestion that it might serve as a healthier alternative to red meat due to its lower content of saturated fats and other harmful compounds [2, 6, 7, 8]. However, other evidence indicates that white meat consumption, when occurring alongside red meat intake, may not independently reduce CVD risk [4]. This discrepancy highlights the complexity of dietary influences on cardiovascular health and the challenges of isolating the effects of individual food groups [9].

The relationship between meat consumption and CVD is further complicated by variations in study design, population characteristics, and the control of confounding factors [10]. Recent systematic reviews and meta‐analyses have debated the differential effects of red and white meat on cardiovascular outcomes [11], emphasizing the need for comprehensive analyses that consider broader dietary patterns, lifestyle factors, and socioeconomic status. For instance, a literature review highlighted the inconsistencies in the literature, suggesting that the observed associations might be influenced by factors beyond meat type alone [8]. Such debates have fueled ongoing discussions about optimal dietary recommendations for reducing cardiovascular risk.

To address these issues, the present study examines the associations between red and white meat consumption and CVD incidence at a population level using an ecological approach. We utilized country‐specific data from United Nations agencies to ensure international comparability. Red meat intake, based on FAO classifications [12], included beef, veal, lamb, mutton, goat, and pork. White meat intake included poultry sources such as chicken, turkey, duck, goose, and guinea fowl. Fish, seafood, rabbit meat, and processed meat products were excluded.

The primary outcome variable is the CVD incidence rate [13], and the main independent variables are the per capita supplies of red meat and white meat [14]. To account for potential confounding effects, we incorporated four established risk factors into our analysis: ageing [15]; socioeconomic status [16, 17]; obesity [18, 19, 20, 21]; and urbanization [16, 22, 23]. By integrating these diverse data sources and addressing both well‐established and contentious aspects of meat consumption, our study aims to provide a comprehensive understanding of the dietary determinants of CVD. This analysis not only builds upon existing research but also situates our findings within the broader scientific discourse on nutrition and cardiovascular health, offering valuable insights that may inform public health recommendations and dietary guidelines.

## 2 Methods

This ecological study employed country level data from multiple United Nations agencies to examine associations between red meat and white meat consumption and CVD incidence. Publicly available, standardized global health and nutrition indicators were used to ensure consistency and comparability across countries. The primary outcome variable, CVD incidence, was sourced from the Institute for Health Metrics and Evaluation (IHME) for 2017. Key independent variables, red meat and white meat supply (kg per capita per year) were extracted from the Food and Agriculture Organization (FAO) Food Balance Sheets for 2017 and served as proxies for national consumption levels.

To account for potential confounding, we included several variables known to influence CVD risk. Ageing was indexed by life expectancy at age 65, obtained from the United Nations Population Division (2017). Socioeconomic status was measured using per capita gross domestic product (GDP) in purchasing power parity (PPP) terms, sourced from the World Bank (2017). Obesity prevalence was defined as the percentage of the adult population (aged 18 and older) with a body mass index (BMI) of 30 kg/m² or greater, obtained from the World Health Organization (WHO) Global Health Observatory (2017). Urbanization was represented by the percentage of the population living in urban areas, sourced from the World Bank (2017).

Ethical approval was not required because the study utilized only nonidentifiable, pre‐existing data.

### 2.1 Statistical Analysis

**Figure 1:**
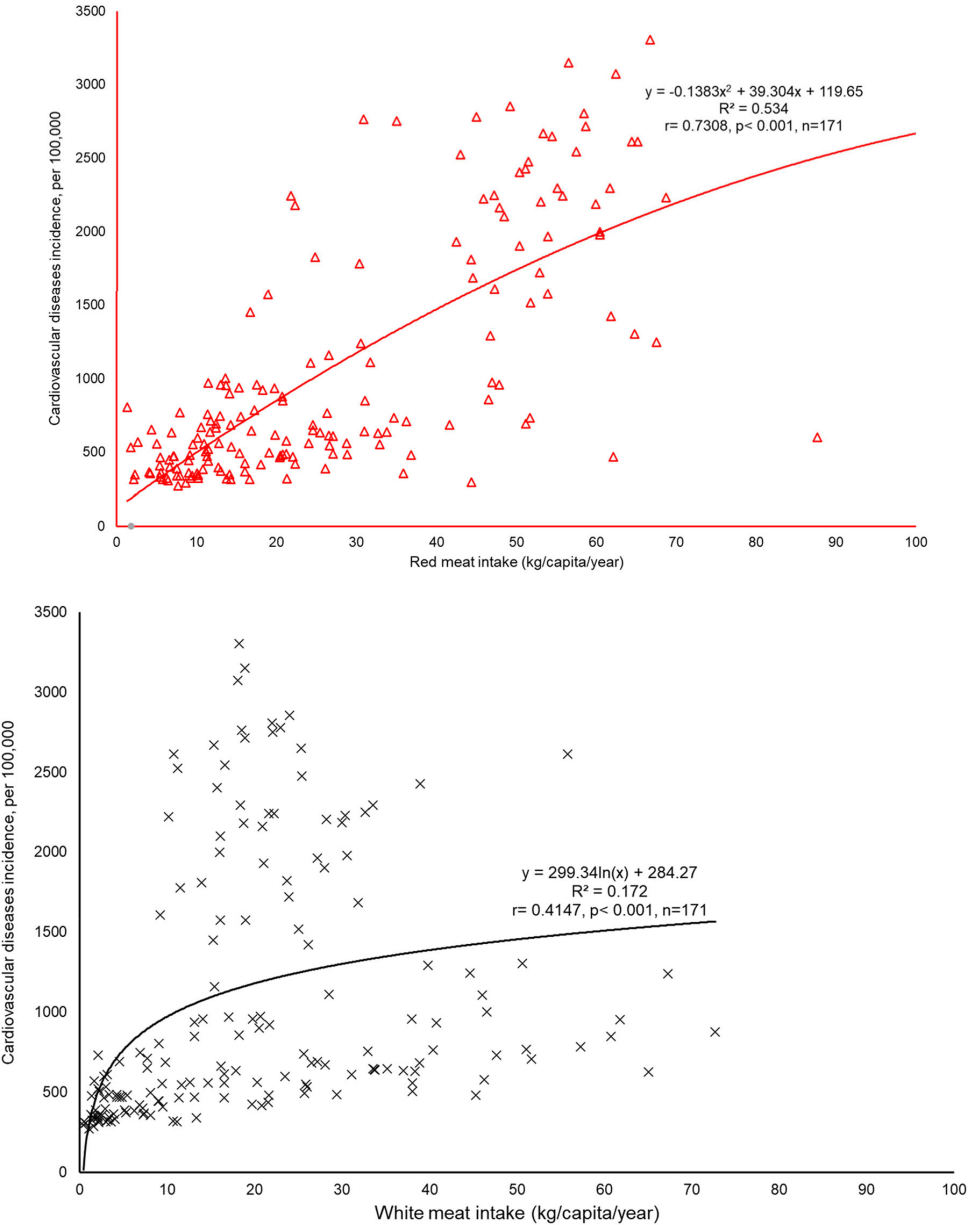
The predicting effects of red meat and white meat on cardiovascular disease incidence respectively. *Figure 1‐1: The relationship between red meat access and cardiovascular disease incidence. Figure 1‐2: The relationship between white meat access and cardiovascular disease incidence*. Data source and definition: Red meat and white, measured with total meats supply quantity (kg/capita/year), the Food and Agriculture Organization 2017; Cardiovascular disease incidence, the number of new cases per 100,000, the Institute for Health Metrics and Evaluation 2017.

We used a multi‐step statistical approach to examine the relationships between meat consumption and CVD incidence. Based on previous publications [24, 25, 26, 27, 28, 29], the roles of red meat and white meat in predicting CVD incidence were examined in four statistical models. Scatter plots (Figure 1) showed that both red meat and white meat correlated with CVD incidence. Next, we conducted a bivariate correlation analysis using Pearson's correlation coefficients and nonparametric correlations to assess the individual associations between red meat, white meat, and CVD incidence. Then, we performed a partial correlation analysis to evaluate the independent effects of red and white meat consumption on CVD while controlling for ageing, socioeconomic status, obesity, and urbanization. Finally, we applied a stepwise linear regression model to identify the strongest predictors of CVD incidence, ranking variables according to their statistical significance and explanatory power.

This comprehensive methodological framework, integrating robust data sources and rigorous statistical analyses, provides a solid foundation for understanding global dietary patterns and their relationship with CVD incidence while acknowledging the inherent constraints of using population level data.

We conducted regression diagnostic analyses to examine multicollinearity among six predictors. In the first model, the predictors included red meat intake, CVD incidence, ageing (e(65)), GDP PPP, obesity prevalence, and urbanization. In the second model, white meat intake was analysed alongside the same set of covariates. The dependent variables were dementia sex disparity and female dementia incidence, respectively. All tolerance values exceeded 0.10, and all variance inflation factor (VIF) values were below 10, indicating that multicollinearity was not a concern [30]. These findings suggest that the predictors contributed independently to the models, supporting the robustness and interpretability of the regression analyses. The absence of significant multicollinearity enhances the reliability of the findings and strengthens the conclusions regarding the relationships among meat intake, demographic and genetic factors, and CVD outcomes.

## 3 Results

Scatter plots (Figure 1) revealed that both red meat and white meat correlated to CVD incidence significantly (*r* = 0.7308, *p*< 0.001, *r* = 0.4147, *p*< 0.001 respectively). Raw data on these three variables were applied for the scatter plots, there was no outlier observed in both plots (Figure 1).

Pearson's *r* correlation analysis model revealed that both red meat and white meat were in significant correlation to CVD incidence (*r* = 0.686, *p* < 0.001 and *r* = 0.552, *p* < 0.001 respectively) (Table 1). Fisher's *r* to *z* transformation revealed that red meat was in a significantly stronger correlation to CVD incidence than white meat (*z* = 2.01, *p* < 0.05). The similar significant correlations of red meat and white meat to CVD incidence were also observed in nonparametric analysis model (*r* = 0.710, *p* < 0.001 and *r* = 0.575, *p* < 0.001 respectively). Fisher's *r* to *z* transformation showed that red meat was in a significantly stronger correlation to CVD incidence than white meat (*z* = 2.13, *p* < 0.05).

**Table 1:** Bivariate (Pearson's *r* and nonparametric) correlation matrix between all variables.

	Red meat	White meat	CVD incidence	Ageing (e_(65)_)	GDP PPP	Obesity %	Urbanization
Red meat	1	0.446***	0.686***	0.591***	0.630***	0.436***	0.449***
White meat	0.429***	1	0.552***	0.635***	0.690***	0.665***	0.566***
CVD incidence	0.710***	0.575***	1	0.759***	0.734***	0.428***	0.526***
Ageing (e_(65)_)	0.609***	0.630***	0.781***	1	0.807***	0.369***	0.556***
GDP PPP	0.678***	0.652***	0.775***	0.816***	1	0.502***	0.716***
Obesity	0.365***	0.654***	0.473***	0.401***	0.483***	1	0.546***
Urbanization	0.481***	0.528***	0.555**	0.602***	0.752***	0.584***	1

*Note:* Pearson *r* (above diagonal) and nonparametric Spearman's *ρ* (below diagonal) correlations are reported. All *p* values are ***p < 0.001. Fisher *r*‐to‐*z* transformation calculated a value of *z* that assessed the significance of the difference between two correlation coefficients, CVD incidence between red meat and white meat respectively: Red meat versus white: *z* = 2.01, *p* < 0.05 in Pearson's model; *z* = 2.13, *p *< 0.05 in nonparametric model.

In almost all instances, people consume a combination of red and white meat, which may substantially contribute to CVD development. A partial correlation analysis model suggested that red meat remained a significant predictor of CVD incidence (*r* = 0.393, *p* < 0.001), however white meat showed nearly negligible correlation to CVD incidence (*r* = −0.046, *p* = 0.567); ageing, GDP PPP, obesity and urbanization were statistically controlled in both analyses (Table 2). This significant difference between the roles of red meat and white meat in predicting CVD incidence remained in Fisher r‐to‐z transformation (*z* = 4.04, *p* < 0.001). When red meat and white meat were alternated together with ageing, GDP PPP, obesity and urbanization, red meat was still in significant correlation to CVD (*r* = 0.391, *p* < 0.001), while white meat showed almost no correlation to CVD incidence (*r* = 0.006, *p* = 0.937) (Table 2).

**Table 2:** Partial correlations for red and white meat in predicting CVD incidence.

Variables	Partial correlation to CVD incidence			Partial correlation to CVD incidence			Partial correlation to CVD incidence		
	*r*	*p*	df	*r*	*p*	df	*r*	*p*	df
Red meat	0.393	< 0.001	156	0.391	< 0.001	155	Also adjusted		
White meat	−0.046	0.567	156	Also adjusted			0.006	0.937	155

*Note:* Both models controlled for confounding factors including ageing (life expectancy at 65 years), GDP PPP, obesity prevalence, and urbanization.

In the standard multiple linear regression (stepwise) analysis, GDP PPP was selected as the variable having the greatest influence on CVD with *R* ^2^ = 0.627, while red meat was placed second increasing *R* ^2^ to 0.686 when red meat, white meat, ageing, GDP PPP, obesity and urbanization were entered as the predicting variables. However, interestingly, the order and *R* ^2^ values remained the same when white meat was not added as a predictor (Table 3). This was another suggestion that white meat was not a significant predictor of CVD, as it did not significantly confound the correlation of red meat to CVD incidence.

**Table 3:** Multiple linear regression stepwise analyses predicting CVD incidence (model A vs. model B).

	White meat not added				White meat added		
Rank	Variable	Adjusted *R* ^2^	Model A: *β* (95% CI)	Rank	Variable	Adjusted *R* ^2^	Model B: *β* (95% CI)
1	GDP PPP	0.627	0.45 (0.33, 0.57)	1	GDP PPP	0.627	0.45 (0.33, 0.57)
2	Red meat	0.686	0.35 (0.22, 0.48)	2	Red meat	0.686	0.35 (0.22, 0.48)
3	Ageing (e_(65)_)	0.711	0.28 (0.15, 0.41)	3	Ageing (e_(65)_)	0.711	0.28 (0.15, 0.41)
	White meat	Not added			White meat	Insignificant	
	Obesity %	Insignificant			Obesity %	Insignificant	
	Urbanization	Insignificant			Urbanization	Insignificant	

*Note:* “Not added” indicates that the variable was not included in Model A, and “Insignificant” indicates that the variable did not contribute significantly when included in Model B.

As white meat intake did not significantly predict CVD incidence, further analysis of the red meat influence on the white meat–CVD relationship was not conducted. Nonetheless, the observed association between white meat and CVD may still be confounded by the stronger effect of red meat intake when both are considered together (Table 3).

## 4 Discussion

CVD is the leading cause of morbidity and mortality owing to multiple aetiologies, including dietary factors such as meat consumption [31]. Extensive studies have consistently correlated red meat consumption with CVD. In contrast, white meat has rarely been singled out for examining its correlation to CVD specifically. By assessing the roles of red meat and white meat in CVD pathogenesis, this study complements previous findings suggesting that red meat consumption may be a significant risk factor for developing CVD, while white meat consumption may not be a significant risk factor in developing CVD. The observed association between white meat intake and CVD incidence may reflect the underlying influence of red meat intake, given the stronger and more consistent correlations identified for red meat consumption (Table 3). This finding may help explain the relative lack of studies reporting on the impact of white meat on CVD.

Of the studies that have investigated the role of white meat in CVD development, the conclusions have been controversial and circumstantial [8, 11, 32]. The rationale for these mixed results may be explained by the differing levels of CVD‐associated substances present in red and white meat. Red meat's effect on CVD has primarily been attributed to substances such as saturated fat [33, 34, 35, 36], l‐carnitine [36, 37], trimethylamine‐N‐oxide (TMAO) [36], heme iron [38] and sodium [39, 40]. These compounds are also present in white meat, but in much lower amounts. For example, red meat contains around four times as much carnitine as white meat [41]. The lower levels in white meat may be insufficient to produce a detectable correlation with CVD prognosis. Similarly, the lower overall consumption of white meat [14] may further mask any potential impact. Supporting this finding is a study showing that red and white meat have identical effects on cholesterol elevations when the subgroups have equivalent saturated fat levels [32]. This was also evidenced in a recent study where red and white meat did not differ significantly in their effects on gut flora in both obese and nonobese females [42]. Other factors such as data collection errors and variations in food preparation may also contribute to these controversial and circumstantial results. For instance, red meat has a stronger flavor due to its higher saturated fat and salt content, which stimulates the taste buds more than white meat. When cooking white meat, individuals often add more seasonings to meet taste preferences, and these seasonings, which contain fats and salt, have been associated with CVD [43]. Moreover, a substantial portion of white meat is consumed on the bone (e.g., drumsticks and wings), making it difficult for subjects to accurately recall the actual weight consumed.

Our ecological analysis, based on country‐level data from 2017, sourced from United Nations agencies, supports other studies associating red meat consumption and CVD incidence at the population level. In bivariate analyses, both red meat and white meat showed significant correlations with CVD incidence; however, once we controlled for key confounders such as ageing, socioeconomic status, obesity prevalence, and urbanization, the association for white meat diminished to near insignificance. This finding suggests that the relationship between white meat and CVD observed in simple correlations is likely confounded by the co‐consumption of red meat, rather than reflecting a direct or modifying effect.

Our study reinforces previous findings that implicate red meat as a substantial risk factor for CVD, potentially due to its higher levels of saturated fats, L‐carnitine, heme iron, and sodium, while white meat, which generally contains lower amounts of these substances, does not independently drive CVD risk when analysed alongside red meat and other confounders [10]. We clarify that our data do not support a direct causal interaction in which red meat “affects” the relationship between white meat and CVD; rather, red meat's robust association with CVD overshadows any potential independent contribution of white meat at the population level.

In addition to these findings, we acknowledge the importance of exploring strategies to mitigate cardiovascular risks associated with meat consumption. Although our study was designed as a global ecological analysis to assess population‐level associations, discussing potential dietary interventions can enhance the practical implications of our findings. Evidence suggests that dietary substitution by replacing red meat with alternative protein sources such as poultry, fish, legumes, nuts, and other plant‐based proteins can favorably influence cardiovascular health [10, 44]. Diets like the Mediterranean diet, which emphasize these alternatives, have been consistently associated with reduced CVD risk [45, 46]. Furthermore, adopting healthier cooking methods and minimizing the consumption of processed meats could further reduce risk factors associated with cardiovascular diseases [47, 48].

Since our study employs an ecological design, we are unable to draw conclusions about individuals or assess intermediate biomarkers like lipid profiles and inflammatory markers [49]. Future research incorporating detailed dietary recalls and biomarker analyses would be invaluable in illustrating the physiological mechanisms underlying these associations and in evaluating the impact of dietary interventions on intermediate outcomes [50]. Moreover, while our current analysis did not specifically explore potential synergistic or antagonistic interactions between red and white meat consumption, we acknowledge that dietary habits typically involve the simultaneous consumption of multiple food groups. Detailed examination of such interactions was not feasible given the limitations of population level data; however, we recommend that future research incorporate individual level data, such as detailed dietary recalls and biomarker assessments, to more effectively explore these interactions [51].

Emerging evidence also suggests that excessive red meat consumption may be implicated in oncogenic risk factors, including the development of solid tumors such as bladder carcinoma. For instance, Barone et al provided insight into the mechanisms by which carcinogenic compounds, formed during processing or cooking, might contribute to oncogenesis [52]. Although a detailed exploration of these oncogenic pathways was beyond the scope of our current ecological study, integrating such findings in future research could provide a more comprehensive understanding of the broader health impacts of meat consumption [53].

Furthermore, the potential role of red meat consumption in modulating circulating androgen levels and consequently influencing the tumor microenvironment in androgen‐dependent cancers such as prostate cancer warrants exploration. Recent evidence suggests that androgen signals may enhance inflammation and promote tumorigenic activation within the tumor microenvironment [54]. Although our current study focused on the cardiovascular implications of meat consumption using ecological data, we recognize that the mechanisms by which red meat may contribute to oncogenic processes, including androgen‐mediated pathways, merit further investigation. While a detailed examination of these mechanisms falls outside the scope of our analysis, incorporating this perspective enriches the broader context of the impact of red meat on health and emphasizes the need for future studies that integrate individual‐level data and biomarker assessments [11].

## 5 Conclusion

This ecological study using population‐level data demonstrates that red meat consumption is significantly associated with increased CVD incidence at the population level, whereas the apparent association for white meat is largely confounded by red meat intake. Our findings indicate that white meat does not independently predict CVD risk when key confounders are accounted for. Although these results highlight the importance of reducing red meat intake and adopting alternative protein sources, they should be interpreted with caution due to the limitations inherent in ecological studies. Future research using individual‐level data and biomarker assessments is needed to validate these associations and explain underlying physiological mechanisms, ultimately informing targeted public health interventions and nutritional guidelines.

## 6 Strength and Limitation

This study has several notable strengths. By leveraging country‐level data from multiple international professional agencies, we were able to construct a comprehensive and globally representative data set. Our methodological approach is robust, employing various statistical models, including bivariate correlations, partial correlations, and stepwise linear regression, to assess the independent contributions of red and white meat consumption to CVD incidence. We controlled for key confounding factors such as ageing, socioeconomic status, obesity prevalence, and urbanization, which bolsters the reliability of our findings. Furthermore, our discussion situates the results within a broader context by addressing potential dietary interventions, exploring possible oncogenic risks, and considering androgen‐mediated pathways, thereby emphasizing the translational relevance of our work in public health.

This study has several limitations that should be acknowledged. First, the analysis did not control for potential confounding factors such as individual lifestyle habits (e.g., smoking, physical activity, alcohol consumption), genetic predisposition, and food preparation methods, all of which may independently or jointly influence CVD risk. Second, the use of population‐level (ecological) data limits the ability to infer causality or draw conclusions about individual dietary behaviors and CVD risk. Consequently, the findings are susceptible to ecological fallacy, where associations observed at the group level may not accurately reflect relationships at the individual level. While ecological studies offer valuable insights into large‐scale patterns, caution is needed when interpreting these associations in an individual context.

In addition, although collinearity diagnostics indicated acceptable variance inflation factor (VIF) values, strong correlations among red meat intake, white meat intake, GDP PPP, and ageing (e(65)) may still influence the interpretation of the regression models. Therefore, caution is also warranted when considering the independent effects of these predictors.

Beyond dietary influences, emerging evidence highlights a complex interplay between cardiovascular disease and cancer, suggesting the presence of shared biological pathways such as chronic inflammation, metabolic dysregulation, and oxidative stress [52, 55]. These overlapping mechanisms underscore the multifactorial nature of CVD development and suggest that future research should integrate a broader perspective encompassing not only lifestyle and dietary factors but also oncological and metabolic processes.

Overall, this study provides valuable insights into global dietary patterns and their association with CVD incidence, while also highlighting the need for future research to confirm these associations and to fully elucidate the underlying mechanisms.

## Author Contributions

**Wenpeng You:** conceptualization, investigation, writing – original draft, methodology, visualization, writing – review and editing, software, formal analysis, project administration, data curation, resources. **Shuhuan Feng:** conceptualization, investigation, methodology, writing – review and editing, project administration, data curation, resources. **Frank Donnelly:** conceptualization, investigation, methodology, visualization, writing – review and editing, formal analysis, data curation, resources.

## Ethics Statement

Human data involved in this study carries only negligible risk and these data are existing that contains only non‐identifiable data about human beings. Therefore, the Human Research Ethics Committee the University of Adelaide has exempted from ethical review (Reference 35404).

## Conflicts of Interest

The authors declare no conflicts of interest.

## Transparency Statement

The lead author Wenpeng You, Shuhuan Feng affirms that this manuscript is an honest, accurate, and transparent account of the study being reported; that no important aspects of the study have been omitted; and that any discrepancies from the study as planned (and, if relevant, registered) have been explained.

## Data Availability

The authors confirm that the data supporting the findings of this study are publicly available from the original sources. The cardiovascular disease incidence data were obtained from the Institute for Health Metrics and Evaluation (IHME) Global Burden of Disease (GBD) database (2017). Red and white meat supply data were extracted from the Food and Agriculture Organization (FAO) Food Balance Sheets (2017). Additional data on ageing, GDP PPP, obesity prevalence, and urbanization were sourced from the United Nations Population Division (2017) and The World Bank (2017). All data sources are cited within the article.
